# Can spirometric norms be set using pre- or post- bronchodilator test results in older people?

**DOI:** 10.1186/1465-9921-13-102

**Published:** 2012-11-16

**Authors:** Bernet Kato, Amund Gulsvik, William Vollmer, Christer Janson, Michael Studnika, Sonia Buist, Peter Burney

**Affiliations:** 1 Respiratory Epidemiology and Public Health, National Heart and Lung Institute, Imperial College, London, UK; 2 Institute of Medicine, University of Bergen, Bergen, Norway; 3 Kaiser-Permanente Center for Health Research, Portland, Oregon, USA; 4 Department of Medical Sciences, Uppsala University, Uppsala, Sweden; 5 Paracelsus Medical University, Salzberg, Austria; 6 Oregon Health and Sciences University, Portland, Oregon, USA

**Keywords:** Normal values, BOLD study, European population

## Abstract

**Background:**

Chronic Obstructive Pulmonary Disease (COPD) is defined by post-bronchodilator spirometry. Data on “normal values” come predominantly from pre-bronchodilator spirometry. The effects of this on diagnosis are unknown.

**Methods:**

Lower limits of normal (LLN) were estimated from “normal” participants in the Burden of Obstructive Lung Disease (BOLD) programme. Values separately derived using pre- and post-bronchodilator spirometry were compared. Sensitivity and specificity of criteria derived from pre-bronchodilator spirometry and pre-bronchodilator spirometry adjusted by a constant were assessed in the remaining population. The “gold standard” was the LLN for the post-bronchodilator spirometry in the “normal population”. For FEV1/FVC, sensitivity and specificity of criteria were also assessed when a fixed value of < 70% was used rather than LLN.

**Results:**

Of 6,600 participants with full data, 1,354 were defined as “normal”. Mean differences between pre- and post- bronchodilator measurements were small and the Bland-Altman plots showed no association between difference and mean value. Compared with using the gold standard, however, tests using pre-bronchodilator spirometry had a sensitivity and specificity of detecting a low FEV1 of 78.4% and 100%, a low FVC of 99.8% and 99.1% and a low FEV1/FVC ratio of 65% and 100%. Adjusting this by a constant improved the sensitivity without substantially altering the specificity for FEV1 (99%, 99.8%), FVC (97.4%, 99.9%) and FEV1/FVC (98.7%, 99.5%).

**Conclusions:**

Using pre-bronchodilator spirometry to derive norms for lung function reduces sensitivity compared to a post-bronchodilator gold standard. Adjustment of these values by a constant can improve validity of the test.

## Background

GOLD defines chronic pulmonary obstructive disease (COPD) in terms of post-bronchodilator lung function [1], but the data from which ‘normal values’ are derived are generally based on pre-bronchodilator lung function measures [2,3]. Although some authors have provided local equations based on post-bronchodilator values or assessed differences when using bronchodilators [4-6], there is no general account of the systematic difference between the two measures. As “normal” values are derived from “normal” people without overt pathology, it might be anticipated that the differences would be small and possibly not clinically important.The Burden of Lung Disease (BOLD) Initiative was designed to develop methodology that can be used to estimate the prevalence and economic burden of COPD. BOLD is a survey of COPD among non-institutionalised adults aged 40 years and over [7]. Study participants complete a questionnaire covering respiratory symptoms, health status, activity limitation, and exposure to potential risk factors, and perform pre- and post-bronchodilator spirometry tests.

This analysis uses BOLD data to estimate the differences between pre- and post-bronchodilator measures and the lower limit of normal values (LLN) derived using either pre- or post-bronchodilator lung function measures in the same subjects. We also assessed the extent to which any differences varied by age, body mass index and height for males and females, separately, and the extent of misclassification using criteria based on pre-bronchodilator spirometry. For FEV1/FVC we also looked at the extent of misclassification using criteria based on pre-bronchodilator spirometry and defining airway obstruction by FEV1/FVC<70% rather than <LLN.

## Methods

We used BOLD data from the following European centres: Maastricht (Netherlands), Lisbon (Portugal), Salzburg (Austria), Bergen (Norway), Krakow (Poland), Hannover (Germany), Uppsala (Sweden), Reykjavik (Iceland), Tartu (Estonia) and London (United Kingdom). Centres were selected to ensure that subjects used in the analyses were from a population of predominantly European origin. Spirometry was performed before and 15–60 minutes after inhalation of 200 μg salbutamol through a spacer. For each participant forced expiratory volume in 1 second (FEV_1_), forced vital capacity (FVC), forced expiratory volume in 6 seconds (FEV_6_) were measured before and after bronchodilator use and the corresponding FEV_1_/FVC ratio was calculated.

We selected spirometry data from subjects in good respiratory health (asymptomatic, lifelong non-smokers) using information from a questionnaire. This included subjects who lacked respiratory symptoms (wheezing, phlegm, cough); had no medical diagnosis of asthma, chronic bronchitis, COPD, or emphysema; and denied ever having suffered tuberculosis or lung cancer or having undergone lung resection. This reference population is hereafter refered to as the “normal” population. To generate predicted and lower limit of normal values, each of the variables (FEV_1_, FVC, FEV_6_, FEV_1_/FVC) was entered into a multiple regression model using height, age and body mass index as predictors. In the regression models, age was centred by subtracting 40, body mass index was centred by subtracting 23 whilst height was centred by subtracting the average height (165 centimetres for females and 175 centimetres for males). We modelled separate regression equations for men and women for pre- and post-bronchodilator FEV_1_, FVC, FEV_6_, FEV1/FVC. Non-linear relationships were also investigated by including the square of height and age as predictors in the models. Models were assessed to determine whether addition of quadratic terms for age and height resulted in an improvement in model fit. Likelihood ratio tests were used to compare nested models.

Predicted values and values for the lower limit of normal (LLN) were estimated for each lung function variable using these results. The LLN value for each lung function variable was estimated as LLN value = predicted value −1.645 * S, where S is an estimate of the standard deviation of the residuals and a residual is the difference between observed and predicted lung function. Bland-Altman plots were used to examine the extent of agreement between measured values of pre- and post-bronchodilator ventilatory function variables. Bland-Altman plots were also drawn for LLN values of pre- and post-bronchodilator ventilatory function variables [8].

For each lung function measurement, differences between pre- and post bronchodilator measures were entered into regression models with height, body mass index and age as predictors to investigate whether there was an association between the difference and these predictors. Mean differences between pre- and post bronchodilator ventilatory function were calculated for the observed values, the predicted values and the lower limits of normal together with their 95% limits of agreement.

Further, for each lung function measurement from those who had been excluded because they had a smoking history, a diagnosis of a history of current respiratory symptoms, differences between pre- and post bronchodilator measures were entered into regression models with GOLD class (mild, moderate, severe or very severe) as a predictor to investigate whether there was association between the difference and the GOLD class.

Finally we used the observed post-bronchodilator ventilatory function from those who had been excluded because they had a smoking history, a diagnosis or a history of current respiratory symptoms to estimate the sensitivity and specificity of using the pre-bronchodilator LLN or the pre-bronchodilator LLN corrected by a constant which was the mean difference between the pre- and post-bronchodilator lower limits of normal. These were then compared with a gold standard of the LLN estimated directly from the post-bronchodilator ventilatory function. Further, for FEV1/FVC we also estimated sensitivity and specificity using the fixed value of 70% rather than LLN. In this case we considered the pre-bronchodilator FEV1/FVC or the pre-bronchodilator FEV1/FVC corrected by a constant which was the mean difference between the observed pre- and post-bronchodilator FEV1/FVC. These were then compared to a gold standard of using the post-bronchodilator FEV1/FVC. In addition to sensitivity and specificity we give Youden’s Index, the sum of sensitivity and specificity −1, a summary validity score that has useful properties in some circumstances [9]. All statistical analyses were performed using Stata 12 (Stata Corporation, College Station, TX USA).

Ethical approval for the study was given in each site before starting data collection and all participants signed a consent form after receiving details of the purpose and content of the study.

## Results

Table 1 shows the number (%) of subjects excluded for different reasons. Of 7430 participants 830 were omitted from all analyses because they had inadequate spirometry (746) or because of missing data (84). Of the 6600 remaining 5246 were excluded from the calculation of normal values because they had a relevant diagnosis, they smoked or they complained of respiratory symptoms. These were subsequently used to analyse the sensitivity and specificity of the different criteria. The other 1354 were used to calculate normal values. Using the spirometric classification of COPD based on post-bronchodilator FEV1, the distribution of the 5246 participants (who were excluded from the calculation of normal values) per GOLD class is; 4053 participants have no COPD i.e. FEV1/FVC(%) ≥ 70%, 633 have mild COPD, 461 have moderate COPD, 88 have severe COPD and 11 have very severe COPD.

**Table 1:** Number of subjects excluded*

	**Male**	**Female**	**Total**
**Total in Sample**	3793 (100%)	3637 (100%)	7430 (100%)
Excluded for poor quality lung function (%)	399 (10.5%)	347 (9.5%)	746 (10.0%)
Incomplete information (cannot be classified)	23 (0.06%)	61 (1.7%)	84 (1.1%)
**Total Included**	3371 (88.9%)	3229 (88.8%)	6600 (88.8%)
Excluded from “normal” population on history* (%)	2787 (74.1%)	2459 (69.3%)	5246 (70.6%)
Of whom:			
Ever smoked	2353 (62.0%)	1575 (43.3%)	3928 (52.9%)
Never-smokers with diagnosis	162 (4.3%)	322 (8.9%)	484 (6.5%)
Never-smokers without diagnosis with symptoms	272 (7.2%)	562 (15.5%)	834 (11.2%)
**Included in “normal population”**	584 (15.4%)	770 (21.2%)	1354 (18.2%)

* Ever Smoked; Diagnosis of asthma, chronic bronchitis, emphysema, lung cancer, COPD, tuberculosis, had part of the lung removed; History of wheezing or whistling in the chest in the previous 12 months, cough on most days for as much as 3 months each year, usually bringing up phlegm or usually have phlegm that is difficult to bring up when has no cold, troubled by shortness of breath when hurrying on the level or walking up a slight hill.

Table 2 describes the studied population showing the number of participants included from each BOLD site. Table 3 describes the studied population, including mean values of pre- and post- bronchodilator FEV_1_, FVC, FEV_6_ and FEV_1_/FVC. As expected the “normal” population had slightly higher ventilatory function and was slightly younger. The normal male population was slightly taller than the other male participants.

**Table 2:** Number of participants in the study population from each BOLD site

**Variable**	**Men**	**Women**
**“Normal” population with adequate spirometry**		
Bergen, Norway	57	73
Hannover, Germany	48	68
Krakow, Poland	29	50
Lisbon, Portugal	49	110
London, England	48	72
Maastricht, Netherlands	46	52
Reykjavik, Iceland	77	56
Salzburg, Austria	133	148
Tartu, Estonia	47	78
Uppsala, Sweden	50	63
“Other”* population with adequate spirometry		
Bergen, Norway	242	237
Hannover, Germany	279	235
Krakow, Poland	215	165
Lisbon, Portugal	275	263
London, England	268	275
Maastricht, Netherlands	233	221
Reykjavik, Iceland	320	289
Salzburg, Austria	498	372
Tartu, Estonia	250	216
Uppsala, Sweden	207	186

* Includes smokers and those with respiratory diagnoses or symptoms.

**Table 3:** Summary of the characteristics of the study population

**Variable**	**Men**			**Women**		
	**N**	**Mean**	**SD**	**N**	**Mean**	**SD**
**“Normal” population with adequate spirometry**						
Age (years)	584	56.4	12.1	770	57.9	11.7
Height (cm)	584	176.5	7.8	770	162.2	7.1
pre- BD FEV1 (litres)	584	3.58	0.78	770	2.52	0.56
pre- BD FEV6 (litres)	584	4.55	0.91	770	3.19	0.68
pre- BD FVC (litres)	584	4.76	0.91	770	3.32	0.69
pre- BD FEV1/FVC (%)	584	75.1	6.6	770	75.9	6.1
post-BD FEV1 (litres)	584	3.67	0.77	770	2.58	0.57
post-BD FEV6 (litres)	584	4.55	0.88	770	3.19	0.67
post-BD FVC (litres)	584	4.71	0.88	770	3.29	0.68
post-BD FEV1/FVC (%)	584	77.9	6.3	770	78.5	6.0
“Other”* population with adequate spirometry						
Age (years)	2787	58.7	11.3	2459	58.7	11.7
Height (cm)	2787	175.4	7.4	2459	162.2	7.3
pre- BD FEV1 (litres)	2787	3.19	0.84	2459	2.34	0.63
pre- BD FEV6 (litres)	2787	4.18	0.96	2459	3.03	0.73
pre- BD FVC (litres)	2787	4.43	0.97	2459	3.19	0.75
pre- BD FEV1/FVC (%)	2787	71.5	9.2	2459	72.9	8.6
post-BD FEV1 (litres)	2787	3.29	0.84	2459	2.41	0.63
post-BD FEV6 (litres)	2787	4.21	0.93	2459	3.05	0.71
post-BD FVC (litres)	2787	4.43	0.94	2459	3.08	0.73
post-BD FEV1/FVC (%)	2787	73.9	9.4	2459	75.6	8.9

* Includes smokers and those with respiratory diagnoses or symptoms.

Table 4 shows the coefficients and the explained variance (R^2^) for the prediction equations for the “normal” population. The coefficients for age, age^2^, (where relevant) and height are the same whether estimating the mean or the lower limit of normal. Values are given for each of the four measures of ventilatory function, pre- and post-bronchodilator and for each sex separately. The pre- and post- bronchodilator values for the intercepts are all within 100 mL of each other with the exception of the lower limit of normal for the FEV_1_ in men, where the difference is approximately 125 mL. The pre- and post- bronchodilator values are often very close to each other and for the FEV_6_ the differences are negligible. Differences in the coefficients for age and height are also negligible, with the exception of the values for the FEV_1_/FVC ratio. The prediction equations for the FEV_1_/FVC ratio are relatively poor as is seen by the much lower R^2^ value, but the differences between pre- and post-bronchodilator values are still less than 3%.

**Table 4:** Regression coefficients for lung function values against age, height and body mass index for each sex and for both pre- and post-bronchodilator values

	**Pre/Post Broncho-dilator**	**Sex**	Intercept (mean)*	Intercept (LLN)*	**Age**	**Age squared**	**Height**	**BMI**	**R**^**2**^
FEV1 (L)	Pre	M	4.153	3.353	−0.034		0.039	−0.02	0.61
	Post	M	4.244	3.475	−0.034		0.039	−0.02	0.63
	*Pre*	F	3.123	2.564	−0.028		0.029	−0.009	0.64
	*Post*	F	3.201	2.648	−0.028		0.029	−0.01	0.65
FVC (L)	Pre	M	5.293	4.322	−0.031		0.055	−0.029	0.58
	Post	M	5.247	4.297	−0.031		0.053	−0.03	0.58
	*Pre*	F	3.991	3.287	−0.028		0.043	−0.017	0.62
	*Post*	F	3.944	3.242	−0.027		0.042	−0.016	0.61
FEV6 (L)	Pre	M	5.171	4.272	−0.036		0.054	−0.031	0.64
	Post	M	5.142	4.262	−0.034		0.052	−0.029	0.63
	*Pre*	F	3.892	3.238	−0.030		0.04	−0.016	0.65
	*Post*	F	3.879	3.225	−0.029		0.04	−0.016	0.65
FEV1/FVC (%)	Pre	M	78.407	68.626	−0.102	−0.00359	−0.062		0.19
	Post	M	80.883	71.563	−0.085	−0.00362	−0.048		0.19
	*Pre*	F	77.876	68.782	−0.041	−0.00446	−0.172	0.105	0.17
	*Post*	F	80.818	71.811	−0.013	−0.00532	−0.119		0.18

*for men aged 40 years, with body mass index 23 and 175 centimetres tall and women aged 40 years, with body mass index 23 and 165 centimetres tall.

Table 5 gives the regression coefficients for the difference between observed pre- and post-bronchodilator lung function values (post bronchodilator – pre-bronchodilator values) for the “normal population”. The intercept represents the mean difference between the two measurements at age 40 years, body mass index 23 and average height. For FEV1 these mean levels increased significantly post-bronchodilator (91 ml (95% CI: 64, 117) for men and 77mL (95% CI: 63, 92) for women), for FEV1/FVC the mean levels also increased significantly post-bronchodilator (2.35% (95% CI: 1.76, 2.93) for men and 2.94% (95% CI: 2.43, 3.45) for women. In contrast the FVC fell significantly by 46 mL (95% CI : 7, 86) in men and by 47 mL (95% CI: 21, 72) in women. The regression coefficients for age, body mass index and height show negligible associations which are for the most part not significant. Further, a random intercept model (a regression model with a separate intercept for each centre) showed no evidence of between-centre heterogeneity, suggesting that the results are similar in each of the centres included in the study. For the “;non-normal” population the differences between pre- and post-bronchodilator were: FEV_1_ in women with mild disease was 59mL (95% CI: 9, 109) greater than those with severe disease; FVC in men with severe disease was 95mL (95% CI: 10, 177) greater than those with mild disease; FVC in men with very severe disease was 275mL (95% CI: 60, 490) greater than those with mild disease; FEV_6_ in men with very severe disease was 176mL (95% CI: 20, 332) greater than those with mild disease; FEV_1_/FVC in men with moderate disease was 0.9% (95% CI: 0.29%, 1.52%) greater than those with mild disease.

**Table 5:** Regression of difference between observed pre- and post-bronchodilator lung function values (post bronchodilator – pre-bronchodilator values) in millilitres

	**Men**			**Women**	
	**Covariate**	**Regression coefficient**	**95% confidence interval**	**Regression coefficient**	**95% confidence interval**
FEV1 (mL)	**Intercept** *	90.61	**(64.14, 117.08)**	77.47	**(62.90, 92.04)**
	Age/year	0.033	(−1.07, 1.13)	−0.31	(−1.01, 0.39)
	**Height/cm**	0.23	(−1.47, 1.93)	1.82	**(0.66, 3.00)**
	BMI	−0.15	(−3.66, 3.37)	−0.81	**(−2.57, 0.95)**
FEV6 (mL)	Intercept *	−29.31	(−59.32, 0.71)	−12.95	(−31.88, 5.98)
	**Age/year**	1.47	**(0.22, 2.71)**	0.79	(−0.12, 1.70)
	Height/cm	−1.66	(−3.58, 0.27)	−0.10	(−1.62, 1.42)
	BMI	1.99	(−1.99, 5.97)	0.52	(−1.76, 2.81)
FVC (mL)	**Intercept** *	−46.16	**(−85.73, -6.58)**	−46.65	**(−72.37, -20.94)**
	Age/year	0.15	(−1.49, 1.80)	0.70	(−0.54, 1.93)
	Height/cm	−2.50	(−5.04, 0.04)	−1.56	(−3.62, 0.51)
	BMI	−0.35	(−5.60, 4.90)	0.26	(−2.85, 3.36)
FEV1/ FVC (%)	Intercept *	2.35	**(1.76, 2.93)**	2.94	**(2.43, 3.45)**
	Age/year	0.016	(−0.008, 0.040)	−0.007	(−0.031, 0.018)
	**Height/cm**	0.015	(−0.022, 0.053)	0.064	**(0.023, 0.105)**
	BMI	0.036	(−0.041, 0.113)	−0.017	**(−0.078, 0.045)**

*Difference at age 40 years, body mass index 23 and 175 centimetres height in men or 165 centimetres height in women.

 Table 6 shows the mean differences and 95% limits of agreement for the observed values, the predicted values and the lower limits of normal. The mean observed differences and the predicted differences are equal, by definition, but the limits of agreement are much narrower for the predicted values, as expected.

**Table 6:** Pre- and post- bronchodilator differences (in millilitres) between observed spirometric values, predicted values and lower limits of normal values for men and women

	** Men**			**Women**	
		**Mean difference**	**95% limits of agreement**	**Mean difference**	**95% limits of agreement**
FEV1 (L)	Observed	91	(−203, 385)	64	(−145, 273)
	Predicted	91	(88, 94)	64	(34, 95)
	LLN	121	(118, 124)	69	(39, 101)
FEV6 (L)	Observed	−0.37	(−338, 337)	3.1	(−267, 273)
	Predicted	−0.37	(−53, 52)	3.1	(−17, 23)
	LLN	18	(−31, 71)	3	(−15.7, 22.7)
FVC (L)	Observed	−49	(−491, 393)	−29	(−396, 338)
	Predicted	−49	(−89, 9)	−29	(−61, 3)
	LLN	−28	(−68,12)	−27	(−59, 5)
FEV1/FVC (%)	Observed	2.76	(−3.73, 9.25)	2.59	(−4.73, 9.91)
	Predicted	2.76	(2.41, 3.11)	2.59	(1.27, 3.91)
	LLN	3.22	(2.87, 3.57)	2.68	(1.36, 4.00)

Table 7 gives the sensitivity and specificity of using the pre-bronchodilator values and the pre-bronchodilator values adjusted with a fixed constant when compared with the gold standard of using the calculated lower limits of normal using the post-bronchodilator values. The data used to estimate these come from the participants excluded from the “normal” population, and therefore do not include individuals who were used to estimate the original norms. Compared with the “gold standard” of the lower limit of normal derived from post-bronchodilator spirometry, use of the pre-bronchodilator LLN criterion results in a substantially lower sensitivity when judging an abnormal FEV1 (sensitivity =78.4%) or FEV1/FVC ratio (sensitivity = 65%), though specificity remains high. Changing the LLN based on the pre-bronchodilator spirometry with an added constant improves the characteristic of the tests overall and the Youden’s index is above 0.95 for all measures. Table 8 gives the sensitivity and specificity of using the pre-bronchodilator values and the pre-bronchodilator values adjusted with a fixed constant when compared with the gold standard of using the post-bronchodilator values when using the fixed cut off of 70% as the criterion for a low FEV1/FVC. In this case adding a constant lowers Youden’s Index and sensitivity, but increases the specificity of the test.

**Table 7:** Sensitivity and Specificity of using the pre-bronchodilator lower limit of normal and using the pre-bronchodilator lower limit of normal with an added constant compared with the lower limit of normal derived from the post bronchodilator values

	**<LLN using pre-bronchodilator**			**<LLN using pre-bronchodilator + K**		
	**Sensitivity**	**Specificity**	**Youden’s index**	**Sensitivity**	**Specificity**	**Youden’s index**
FEV1	78.4%	100%	0.78	99%	99.8%	0.99
FEV6	96.1%	100%	0.96	98%	99.8%	0.98
FVC	99.8%	99.1%	0.99	97.4%	99.9%	0.97
FEV1/FVC	65.0%	100%	0.65	98.7%	99.5%	0.98

Results for 5246 subjects in the “non-normal” survey population. The participants included were those with adequate spirometry, but excluded from the estimation of normal values because of a history of smoking or of respiratory diagnosis or symptoms.

Values of “K”.

for FEV_1_ (mLs): 121 (men); 69 (women).

for FEV_6_ (mLs): 18 (men); 3 (women).

for FVC (mLs): -29 (men); -27 (women).

for FEV_1_/FVC (%) : 3.22 (men); 2.68 (women).

**Table 8:** Sensitivity and Specificity of using the pre-bronchodilator and using the pre-bronchodilator with an added constant compared with the post-bronchodilator values

	**<70% using pre-bronchodilator**			**<70% using pre-bronchodilator + K**		
	**Sensitivity**	**Specificity**	**Youden’s index**	**Sensitivity**	**Specificity**	**Youden’s index**
FEV1/FVC	90%	85%	0.75	79%	94%	0.73

Results for 5246 subjects in the “non-normal” survey population. The participants included were those with adequate spirometry, but excluded from the estimation of normal values because of a history of smoking or of respiratory diagnosis or symptoms.

Values of “K”: 2.76 (men); 2.59 (women).

**Figure 1:**
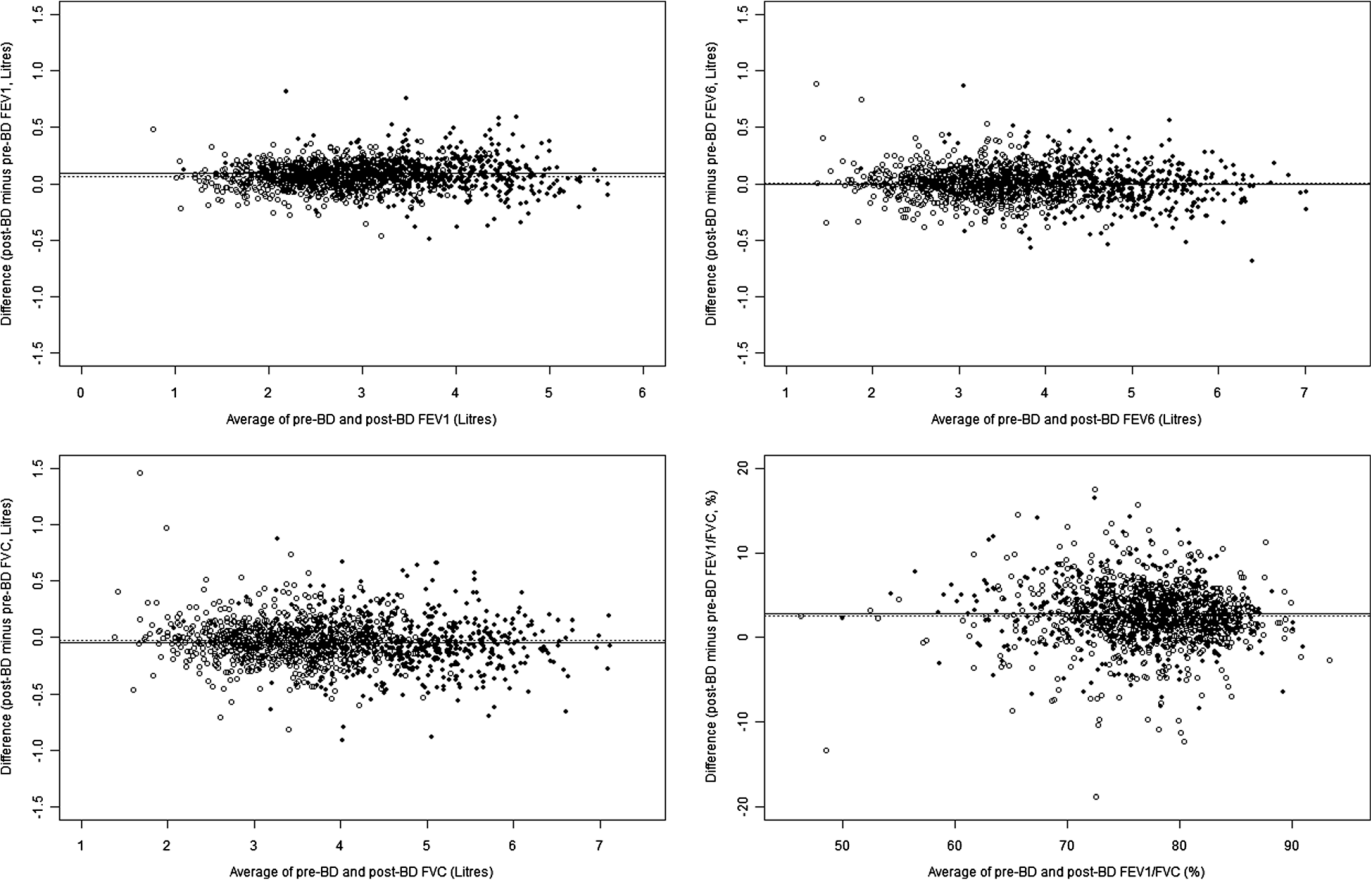
**Bland Altman plots for observed FEV1, FEV6, FVC and FEV1/FVC (males – solid circles, females – hollow circles).** The lines show the mean difference between pre- and post bronchodilator measured lung function for males (solid line) and females (dotted line).

Bland-Altman plots for the two sexes show no obvious relation between the difference and magnitude of FEV_1_, FEV_6_, FVC and FEV_1_/FVC (%), respectively. (Figure 1). The mean differences between pre- and post-bronchodilator values for measured FEV1, FVC, FEV6 and FEV1/FVC are 76 mL (95% CI 69, 83 ), -38 mL (95% CI −48, -27), 1.6 mL (95% CI −6.6, 9.8 ) and 2.66% (95% CI 2.47, 2.85), respectively.

**Figure 2:**
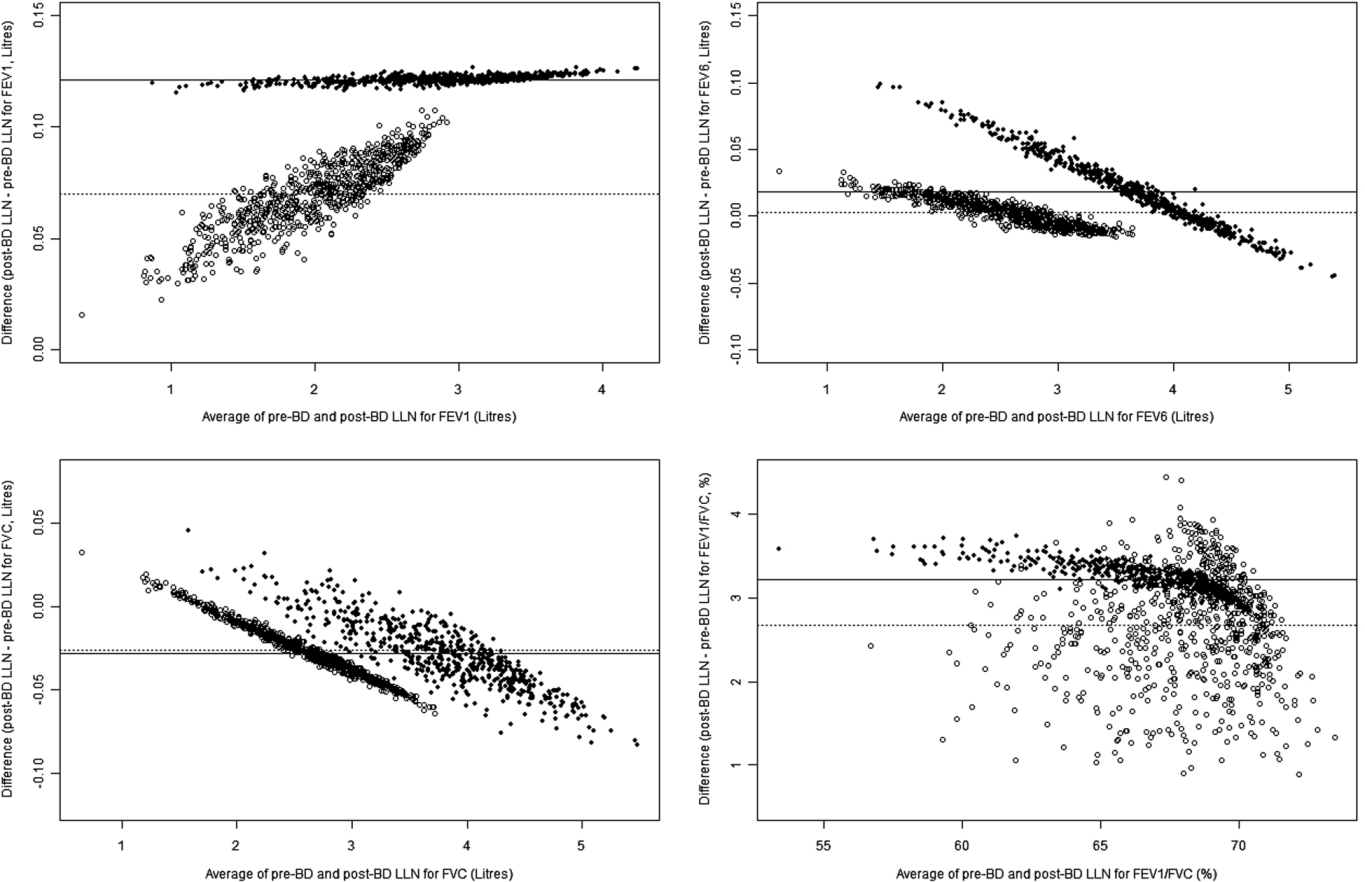
**Bland Altman plots for LLN values of FEV1, FEV6, FVC and FEV1/FVC (males – solid circles, females – hollow circles).** The lines show the mean difference between pre- and post bronchodilator LLN values for males (solid line) and females (dotted line).

Figure 2 gives similar plots for the lower limits of normal. The mean differences between pre- and post-bronchodilator values for the LLN of FEV_1_, FVC, FEV_6_ and FEV_1_/FVC are 92 mL (95% CI 91, 94 ), -27 mL (95% CI −28, -26 ), 9.54 mL (95% CI 8.44, 10.64 ) and 2.91% (95% CI 2.88, 2.94), respectively. All of the above differences are adjusted for age, height and sex. It is clear that the distribution of the difference is irregular with respect to the mean value, though the nature of the irregularity is different for each sex and each measurement.

**Figure 3:**
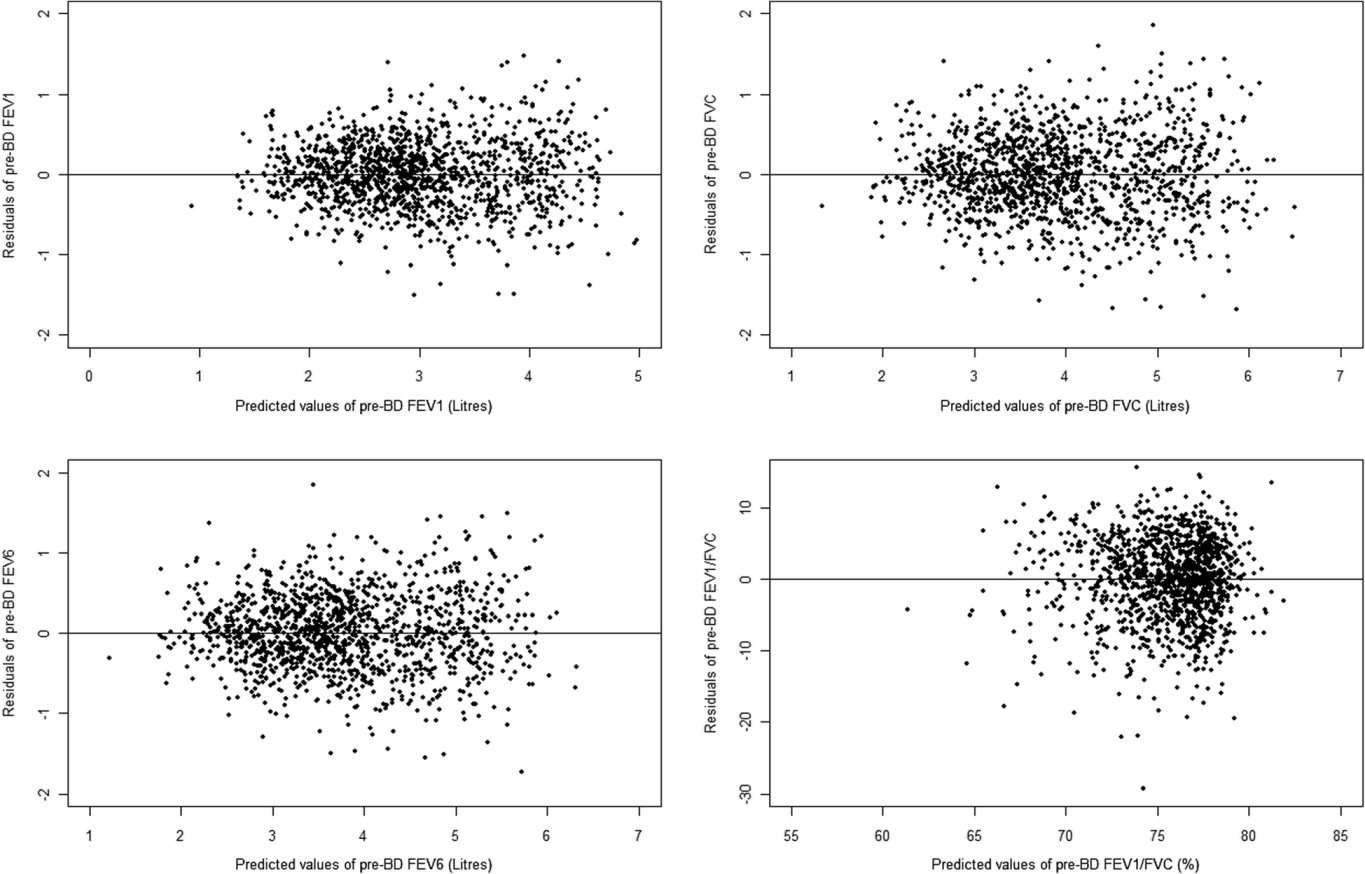
Plots of residuals versus predicted values for pre-bronchodilator lung function.

**Figure 4:**
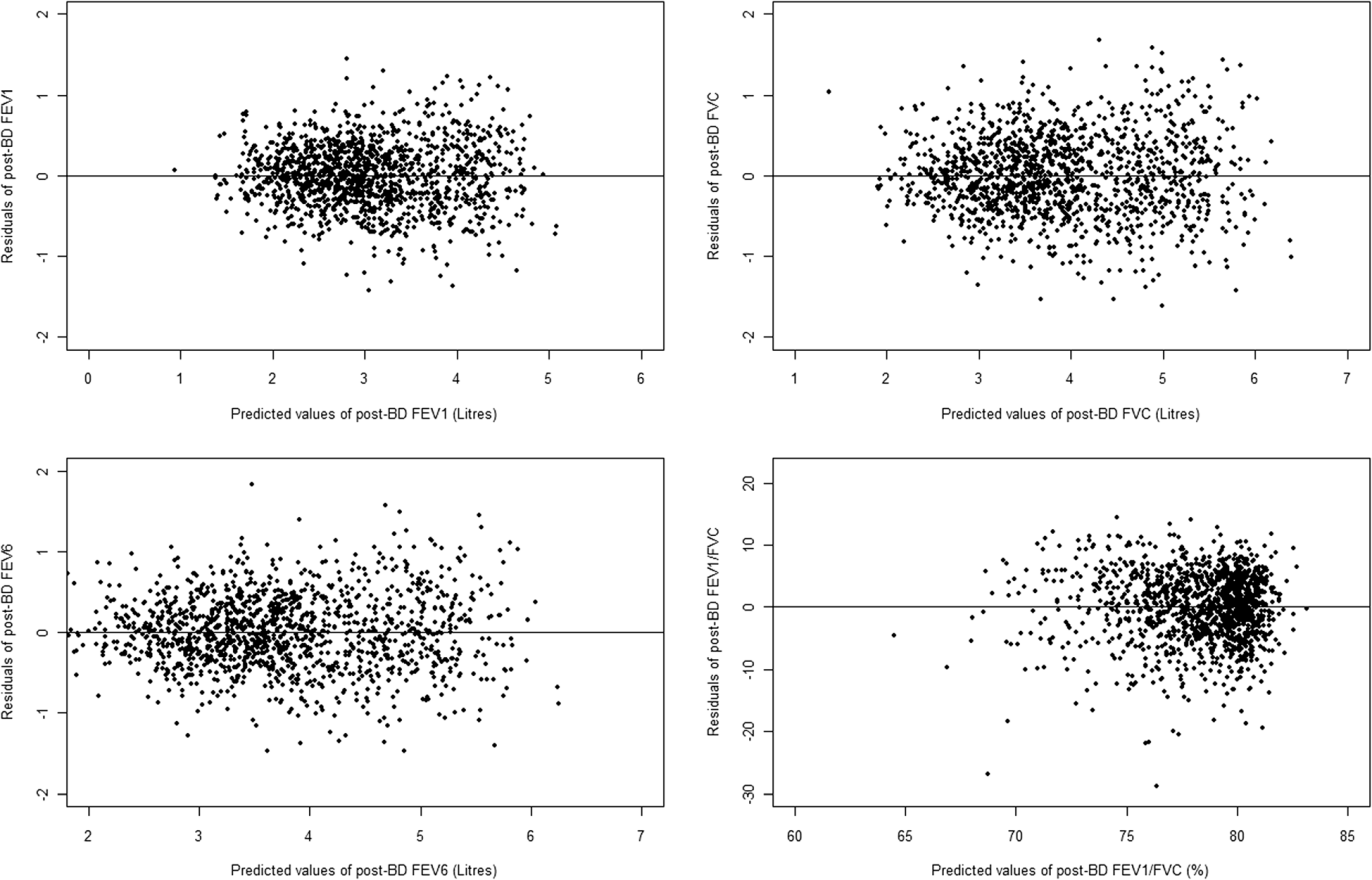
Plots of residuals versus predicted values for post-bronchodilator lung function.

Plots of residuals (Figures 3 and 4) for pre- and post-bronchodilator FEV_1_, FVC, FEV_6_ and FEV_1_/FVC against the corresponding predicted values reveal a pileup of residuals in the centre of the plot at each predicted value and a normal distribution of residuals trailing off symmetrically from the centre. Further the band enclosing the residuals is approximately equal across the range of predicted values). This shows that the assumptions of normality and homoscedasticity are met and implies that it is reasonable to estimate the fifth percentile of lung function (LLN) of each subject by subtracting 1.645*S from a subject’s predicted value.

## Discussion

As expected, the observed value of lung function in a “normal” population changes little with the use of a bronchodilator and differences between centres were not significant. The case for the predicted and for the lower limit of normal is more complicated, although the mean difference is the same for the predicted as for the observed, by definition, and similar in magnitude for the lower limit of normal. This analysis has shown that, although the pre-bronchodilator lower limit of normal for the lung function test is a reasonable approximation to the lower limit of normal, using pre-bronchodilator norms substantially reduces the sensitivity of spirometry in identifying cases of chronic airflow limitation. Addition of a fixed amount provides an even more nearly approximate value to the true post-bronchodilator “normal” values, raising sensitivity to over 99%.

Although it is inconsistent to use pre-bronchodilator “normal values” to assess post-bronchodilator responses the similarity of results using either method is not surprising. Resting tone in the normal airway is low and the effect of a bronchodilator is likely to be similarly small. These data come from a study with a very high level of quality assurance and with a strong training programme for the technicians prior to starting the programme.

Estimating predicted values produced a reasonable fit and similar parameter estimates using either the pre- or post- bronchodilator results. Comparing the observed results using either method showed small average differences between pre- and post- bronchodilator results and no discernable variation in the difference with respect to the average of the pre- and post-bronchodilator values.

The changes in lung function observed in the “normal” population following bronchodilator are, as expected, small. The small fall in FVC was not predicted but the size of the fall is small and may be due to tiring of the participants. If so, it is part of the usual post-bronchodilator test which is almost always performed after an initial test without bronchodilator. The Bland-Altman plots for the predicted values (not shown), and hence also the values for the lower limits of normal, are less satisfactory and show variable associations between mean difference and average value for the different measurements. This in part reflects the observation that the difference is not a constant but varies also by age and height (Table 5). Nevertheless the limits of agreement are narrow, for FEV1 lying between ± 3.5 mL (men), and ± 31 mL (women); for FEV6: ±53 mL (men), ±20 mL (women); for FVC ± 40 mL (men), ± 32 mL (women); for FEV1/FVC: ± 0.35% (men) ± 1.32% (women).

The data come from a large multi-centric study of general population samples over the age of 40 years, and the sites chosen were inhabited by people of predominantly European origin. Younger populations have a greater tendency to reversible airway obstruction and although the extent of this in people with neither respiratory symptoms nor respiratory diagnoses is likely to be more limited, we cannot extrapolate our findings to younger age groups. Nor can we extrapolate the findings to other ethnic groups, though again the findings are likely to be similar as there are unlikely to be large variations in resting tone in normal airways in different ethnic groups. Within this population the findings were similar in each of the centres included.

In the BOLD study bronchodilation is achieved by administering 200μg salbutamol via a spacer. The GOLD convention is to give a 400μg dose. The BOLD decision was based on the evidence that 200μg achieves almost as great an effect as 400μg [10] and provides a more acceptable profile of side effects for a population survey of volunteer participants with much less likelihood of having ever used a bronchodilator before. We believe that an additional 200μg of salbutamol would have achieved very little additional change in this group of participants.

In epidemiological studies it is not always necessary or even desirable to divide a population into “diseased” and “healthy”, and ventilatory function can be treated as a continuous variable. However, where a binary variable is required, as it is, for instance, when making a diagnosis, and hence when estimating a prevalence, there is a need to decide on the cut-off point between the normal and abnormal. In many clinical situations this is done by estimating the 95% tolerance limits in the “normal population”. This is however an arbitrary criterion and is agreed by convention. There is no clinical reason for not using a slightly stricter or more relaxed criterion if this is more convenient. There is a great deal of information collected from around the world on “normal” ventilatory function based on this method [11]. The principles underlying this collection have been similar for many years, though the operational definitions of “normal” participants have varied. Nevertheless almost all studies have been conducted without the use of a bronchodilator, which is essential in the use of the test to define COPD according to international guidelines. Given the arbitrary nature of the conventions for defining “normal” values, including the choice of the 95% tolerance limits and the decision on whom to include as “normal” or exclude as potentially abnormal, the differences introduced by using pre- rather than post- bronchodilator ventilatory function, though substantial, are not large. Providing that they are used consistently use of norms based on pre-bronchodilator spirometry are probably acceptable for most purposes. It is, by contrast, very important that the test itself is administered with a bronchodilator, and this is still not the usual practice in prevalence surveys.

In epidemiological studies we recommend the use of the lower limit of normal as the criterion for a low FEV1/FVC ratio as this is the most convenient way to adjust prevalence to age. Nevertheless some arguments are still made for the fixed ratio of 70% and this remains the recommendation of GOLD. Mannino has argued that as the lower limit of normal is less sensitive than the fixed ratio, which is the case at least over the age of about 45 years, that its use leads to “under-diagnosis” of true cases [12]. This may be a consideration where over-diagnosis is not a concern. A Dutch study in Primary Care has also reported that the fixed ratio gives a more accurate assessment of disease when compared to clinical opinion in diagnosing clinical COPD [13]. As in other reports the fixed ratio was more sensitive and less specific than the lower limit of normal and in a lower prevalence environment would have come to the opposite conclusion. As the Fixed Ratio is still used we have provided results for this criterion also.

In the absence of a large set of observations on post-bronchodilator ventilatory function, the options for defining “abnormal” values are, effectively, three. First, and easiest, the pre-bronchodilator norms can be used. As bronchodilators have little effect in the absence of smoking, diagnoses and symptoms, this is not a bad approximation and, as the choice of a 95% cut off for the “normal value” is in any case arbitrary, it is adequate at least where only internal comparisons are being made, or where the comparisons are made with studies that have made similar assumptions. Second, the estimates can be changed by a fixed amount to take account of the small average difference induced by the bronchodilator in normal people. As the bronchodilator has slightly different effects on normal subjects according to their age, sex and height, this will not provide a perfect solution and there will be a small distortion in the estimate which will vary by these characteristics. The effect is however small and not of clinical significance when using the lower limit of normal. It should be noted that the small correction added here is based on the BOLD data and needs to be tested in other populations, but it gives some idea of the size of the correction and its ability to adjust adequately for the use of pre-bronchodilator standards. It is notable that the addition of this constant did not improve the results for the test based on the fixed ratio, as judged by Youden’s index. This is likely to be due to the greater distortion induced when adding a constant where age has not been adjusted for. Third, internal estimates of normal values can be estimated from the post-bronchodilator measurements taken in the study itself on “normal” participants. This last option is clearly not available to clinical studies and it has the disadvantage in epidemiological studies that the estimates will almost always be determined on relatively small samples, which makes them inherently unreliable.

## Conclusions

Using different predictors of normal lung function based on pre- or post- bronchodilator spirometric values provides slightly different results, but these may not be of great clinical significance provided they are used consistently in comparative studies. In a clinical setting spirometry should be used in combination with other information to guide management and these small differences are unlikely to be important when setting criteria for a positive screening test if this is based on a pre-bronchodilator assessment of spirometry. We have provided prediction equations for post-bronchodilator lung function in people over the age of 40 years living in Europe. We have also provided approximate mean differences between pre-and post-bronchodilator values and we have given estimates of the effect of using the different methods on sensitivity and specificity of a test for COPD.

## Competing interests

The authors declare that they have no competing interests.

## Authors' contributions

BSK analysed and interpreted the data and wrote the manuscript. PGJB co-ordinated the project and suggested the analysis and wrote the first draft of parts of the introduction and discussion, AG, CJ, MS, all helped to collect data, WV co-ordinated the initial data management and analysis and ASB initiated the BOLD project. All authors reviewed and commented on earlier drafts and read and approved the final manuscript.
